# The *APOB* insertion/deletion polymorphism (rs17240441) influences postprandial lipaemia in healthy adults

**DOI:** 10.1186/s12986-015-0002-9

**Published:** 2015-03-08

**Authors:** Karani Santhanakrishnan Vimaleswaran, Anne M Minihane, Yue Li, Rosalyn Gill, Julie A Lovegrove, Christine M Williams, Kim G Jackson

**Affiliations:** Hugh Sinclair Unit of Human Nutrition, Department of Food and Nutritional Sciences, University of Reading, Reading, RG6 6AP UK; Institute for Cardiovascular and Metabolic Research (ICMR), University of Reading, Reading, UK; Department of Nutrition, Norwich Medical School, University of East Anglia, Norwich, UK; Boston Heart Diagnostics, Framingham, MA 01702 USA

**Keywords:** *APOB* gene, Signal peptide polymorphism, Insertion/deletion polymorphism, Postprandial study, Sequential test meals, Triacylglycerol

## Abstract

**Background:**

Apolipoprotein (apo)B is the structural apoprotein of intestinally- and liver- derived lipoproteins and plays an important role in the transport of triacylglycerol (TAG) and cholesterol. Previous studies have examined the association between the *APOB* insertion/deletion (ins/del) polymorphism (rs17240441) and postprandial lipaemia in response to a single meal; however the findings have been inconsistent with studies often underpowered to detect genotype-lipaemia associations, focused mainly on men, or with limited postprandial characterisation of participants. In the present study, using a novel sequential test meal protocol which more closely mimics habitual eating patterns, we investigated the impact of *APOB* ins/del polymorphism on postprandial TAG, non-esterified fatty acids, glucose and insulin levels in healthy adults.

**Findings:**

Healthy participants (n = 147) consumed a standard test breakfast (0 min; 49 g fat) and lunch (330 min; 29 g fat), with blood samples collected before (fasting) and on 11 subsequent occasions until 480 min after the test breakfast. The ins/ins homozygotes had higher fasting total cholesterol, LDL-cholesterol, TAG, insulin and HOMA-IR and lower HDL-cholesterol than del/del homozygotes (P < 0.017). A higher area under the time response curve (AUC) was evident for the postprandial TAG (P < 0.001) and insulin (P = 0.032) responses in the ins/ins homozygotes relative to the del/del homozygotes, where the genotype explained 35% and 7% of the variation in the TAG and insulin AUCs, respectively.

**Conclusions:**

In summary, our findings indicate that the *APOB* ins/del polymorphism is likely to be an important genetic determinant of the large inter-individual variability in the postprandial TAG and insulin responses to dietary fat intake.

## Findings

### Introduction

Apolipoprotein (Apo)B is the structural apoprotein of intestinally- (e.g. chylomicrons, apoB-48) and liver- (e.g. very low density lipoprotein (VLDL), apoB-100) derived lipoproteins and plays an important role in the transport of triacylglycerol (TAG) and cholesterol [1]. The *APOB* insertion/deletion (ins/del) polymorphism (rs17240441), which produces a difference of three amino acids in the signal peptide, has been associated with fasting total cholesterol (TC) [2,3], TAG [4,5], high density lipoprotein-cholesterol (HDL-C) [2] and low density lipoprotein-cholesterol (LDL-C) [2,3] concentrations, along with cardiovascular disease-related outcomes [6-11]. In addition, the effect of this polymorphism on lipid metabolism appears to be modulated by dietary fat and cholesterol intakes [4,12].

Non-fasting (postprandial) TAG is now recognised as a highly significant and arguably independent risk factor for cardiovascular disease [13-16]. However, the postprandial lipaemic response is highly heterogeneous, with inter-individual variability in response to meal ingestion shown to be modulated by environmental and genetic factors [13,14,17]. Previous studies have examined the association between the *APOB* ins/del polymorphism and postprandial lipaemia in response to a single meal [8,9,18,19] but findings have been inconsistent with studies often underpowered to examine genotype-lipaemia associations, focussed mainly on men, or with limited (both in terms of time-points and measurements) postprandial characterisation of participants. Hence, in the present study, using a novel sequential test meal protocol which more closely mimics habitual eating patterns, we examined the effect of the *APOB* ins/del polymorphism on postprandial TAG, non-esterified fatty acids (NEFA), glucose and insulin levels in healthy adults.

### Methods

#### Study participants

The study was performed using postprandial data from 147 healthy participants who underwent the same sequential test meal protocol and similar inclusion/exclusion criteria, at the University of Reading between 1997 and 2007, as previously described [20]. Briefly, healthy men and women aged 20-70 years, with a body mass index (BMI) between 19-32 kg/m^2^, fasting TAG levels ≤4 mmol/l and total cholesterol ≤8 mmol/l were recruited (Table 1). The studies were approved by the University of Reading Research Ethics Committee and the West Berkshire Health Authority Ethics Committee, and written informed consent was obtained from all participants.

**Table 1:** **Characteristics of the men and women in the postprandial dataset (n = 147)**

	**Men**	**Women**	**P value**
Age (y)	54 ± 10	54 ± 11	0.868
BMI (kg/m^2^)	27.6 ± 3.1	25.6 ± 3.5	*0.002*
TC (mmol/l)	6.15 ± 0.94	5.99 ± 0.97	0.390
TAG (mmol/l)	2.05 ± 0.83	1.39 ± 0.50	*<0.001*
HDL-C (mmol/l)	1.10 ± 0.26	1.41 ± 0.27	*<0.001*
LDL-C (mmol/l)	4.12 ± 0.91	3.95 ± 0.81	0.313
NEFA (μmol/l)	490 ± 185	539 ± 202	0.288
Glucose (mmol/l)	5.32 ± 0.67	5.14 ± 0.48	0.109
Insulin (pmol/l)	54.0 ± 32.0	39.2 ± 26.3	*0.011*
HOMA-IR	2.2 ± 1.5	1.5 ± 1.1	*0.010*

Values represent mean ± SD for n = 112 men and n = 35 women.

The data represents n = 85 men and n = 34 women for insulin and HOMA-IR, and n = 109 men and n = 33 women for NEFA.

*Abbreviations*: *BMI* body mass index, *NEFA* non-esterified fatty acids, *LDL-C* low density lipoprotein cholesterol, *HDL-C* high density lipoprotein cholesterol, *HOMA-IR* homeostasis model assessment of insulin resistance, *TAG* triacylglycerol, *TC* total cholesterol.

#### Postprandial protocol

Details of the postprandial protocol have been described previously [20]. Briefly, study participants were asked to refrain from alcohol or organised exercise regimens on the previous day and were provided with a relatively low fat (<10 g fat) evening meal to standardise short-term fat intake. After a 12 h overnight fast, a blood sample was taken. Following a standard test breakfast (0 min; 3.9 MJ energy, 111 g carbohydrate, 19 g protein and 49 g fat) and lunch (330 min; 2.3 MJ energy, 63 g carbohydrate, 15 g protein and 29 g fat), blood samples were taken at 30–60 min intervals until 480 min after the test breakfast.

#### Biochemical analysis

Plasma lipids and glucose were analysed with an automated analyser (Instrumentation Laboratory (UK) Ltd, Warrington, UK) using enzyme-based kits supplied by Instrumentation Laboratory and Alpha Laboratories (Eastleigh, UK). In the fasting sample, HDL-C was measured in the supernatant following precipitation of the apoB-containing lipoproteins with a dextran-manganese chloride reagent. LDL-C was calculated using the Friedewald formula. Insulin was measured by ELISA (Dako Ltd, High Wycombe, UK). The homeostasis assessment model of insulin resistance (HOMA-IR) was calculated using the formula: [fasting insulin (pmol/l) × fasting glucose (mmol/l)]/135. All samples for each individual were analysed within a single batch and the inter-assay coefficient of variation for the assays were less than 5%.

#### Genotyping

DNA was isolated from the buffy coat layer as previously described [20]. The ins/del polymorphism was detected using a PCR-agarose gel electrophoresis as described previously [4]. The genotype distribution of this polymorphism was in concordance with Hardy-Weinberg equilibrium (P = 0.87).

#### Statistical analysis

Statistical analysis was carried out using the STATA version 13. The data were checked for normality prior to statistical analysis and transformed where necessary. The data in Table 1 which could not be normalised (age, BMI, fasting total cholesterol, glucose, insulin, NEFA and HOMA-IR) were analysed using a Mann-Whitney test and an Independent-Samples *t* test for those with a normal distribution. Postprandial summary measures for the TAG, glucose and insulin responses included area under the curve (AUC, 0-480 min) and incremental AUC (IAUC, 0-480 min). Since postprandial NEFA concentrations show an initial drop after the breakfast meal, NEFA AUC and IAUC responses were calculated from the minimum concentration until the end of the postprandial investigation (120-480 min). Linear regression analysis (adjusting for age, gender and BMI) was used to determine the impact of genotype on the fasting metabolites and postprandial summary measures (AUC and IAUC). The interactions of the polymorphism with age, gender and BMI were tested by including the interaction term in the linear regression model.

### Results

Table 1 shows the characteristics of the men and women in the postprandial cohort. Men had significantly higher BMI, fasting TAG, insulin and HOMA-IR, and lower HDL-C than women (P ≤ 0.010).

Compared with the del/del homozygotes, insertion allele carriers had higher fasting TC (versus ins/del P = 0.017; ins/ins, P = 0.013), LDL-C (versus ins/del P = 0.011; ins/ins, P = 0.017), TAG (versus ins/del, P = 0.049; ins/ins, P = 0.006), and lower HDL-C (versus ins/del, P = 0.004; ins/ins, P = 0.003). Ins/Ins homozygotes had higher fasting insulin and HOMA-IR than the del/del (P = 0.001) and ins/del (P = 0.012) groups (Table 2). There was no association between the polymorphism and fasting NEFA or glucose concentrations (P > 0.079).

**Table 2:** **Fasting metabolites and postprandial measures according to the** ***APOB*** **insertion/deletion polymorphism in the study participants**

**Participant characteristics**	**Del/Del (n = 52)**	**Ins/Del (n = 70)**	**Ins/Ins (n = 25)**	Overall P value*
Age (y)	52 ± 2	56 ± 1	53 ± 2	0.176
Gender (men/women)	36/16	53/17	23/2	0.090
Body mass index (kg/m^2^)	26.6 ± 0.4	27.2 ± 0.4	27.9 ± 0.8	0.184**
***Fasting metabolites***				
Total cholesterol (mmol/l)	5.77 ± 0.12^A^	6.26 ± 0.11^B^	6.43 ± 0.21^B^	*0.004*
Triacylglycerol (mmol/l)	1.62 ± 0.09^A^	1.96 ± 0.10^B^	2.25 ± 0.14^B^	*0.007*
LDL-C (mmol/l)	3.75 ± 0.10^A^	4.24 ± 0.11^B^	4.32 ± 0.19^B^	*0.007*
HDL-C (mmol/l)	1.29 ± 0.04^A^	1.13 ± 0.03^B^	1.04 ± 0.04^B^	*0.002*
NEFA (μmol/l)	525.4 ± 31.5	503.5 ± 22.2	448.0 ± 22.1	0.079
Glucose (mmol/l)	5.23 ± 0.08	5.28 ± 0.07	5.39 ± 0.15	0.798
Insulin^∞^ (pmol/l)	43.9 ± 3.37^A^	48.9 ± 4.08^A^	74.9 ± 12.3^B^	*0.007*
HOMA-IR	1.73 ± 0.14^A^	1.99 ± 0.18^A^	3.15 ± 0.57^B^	*0.004*
***Postprandial summary measures***				
Triacylglycerol (mmol/l × 480 min)				
AUC	1024.3 ± 52.5^A^	1268.9 ± 61.3^B^	1841.6 ± 135.4^C^	*<0.001*
IAUC	273.3 ± 22.4^A^	326.0 ± 25.4^A^	511.6 ± 58.3^B^	*<0.001*
NEFA (mmol/l × 300 min)^¥^				
AUC	160.0 ± 5.8	153.4 ± 6.2	149.8 ± 6.3	0.153
IAUC	94.8 ± 7.3	101.7 ± 3.9	91.3 ± 5.2	0.539
Glucose (mmol/l × 480 min)				
AUC	2979.1 ± 48.9	2946.9 ± 83.9	3184.9 ± 62.4	0.527
IAUC	489.9 ± 43.9	557.7 ± 31.4	533.3 ± 34.7	0.306
Insulin^∞^ (nmol/l × 480 min)				
AUC	25.7 ± 8.6^A^	24.8 ± 6.1^A^	81.5 ± 23.0^B^	*0.036*
IAUC	113.2 ± 21.5	91.2 ± 9.8	172.8 ± 25.4	0.108

Values represent mean ± SEM. *Abbreviations*: *AUC* area under the curve, *HDL-C* high density lipoprotein cholesterol, *HOMA-IR* homeostasis model assessment of insulin resistance, *IAUC* incremental area under the curve, *LDL-C* low density lipoprotein cholesterol, *NEFA* non-esterified fatty acids.

Means with different superscript capital letters (A,B,C) denote significant differences between the Del/Del, Del/Ins and Ins/Ins genotypes (P < 0.05; see text for exact P values).

^∞^Only 119 individuals had data for fasting insulin (n = 52 for del/del, n = 53 for del/ins and n = 14 for ins/ins) and n = 35 with postprandial insulin concentrations (n = 10 for del/del, n = 15 for del/ins and n = 10 for ins/ins).

*Adjusted for age, gender and BMI.

**Adjusted for age and gender.

^¥^AUC and IAUC for the NEFA response are calculated from the time of suppression (120 min) to the end of the postprandial period (480 min).

**Figure 1:**
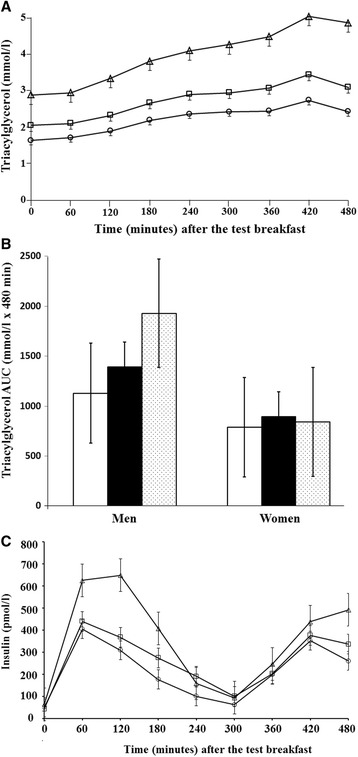
**Effect of APOB ins/del polymorphism on postprandial triacylglycerol (TAG) and insulin response. A**. Mean (SEM) for the postprandial TAG response in the *APOB* del/del (n = 52, open circles), del/ins (n = 70, open squares) and ins/ins (n = 25, open triangles) genotype groups after consumption of a test breakfast (49 g fat) at 0 min and a test lunch (29 g fat) at 330 min. There was a 44% and 31% higher TAG area under the curve (AUC) (del/del vs ins/del, P = 0.010; del/del vs ins/ins, P < 0.001; ins/del vs ins/ins, P < 0.001) in the ins/ins than del/del homozygotes and ins/del heterozygotes, respectively. **B**. Mean (SEM) for the AUC for the postprandial TAG response in men and women in the *APOB* del/del (white bars; n = 36 men/n = 16 women), del/ins (black bars; n = 51 men/n = 17 women) and ins/ins (dotted bars; n = 23 men/n = 2 women) genotype groups. There was a significant effect of genotype on the TAG AUC in men only (P = 0.043, del/del vs ins/del; P < 0.001, ins/ins vs both del/del and ins/del) whereas differences between genotypes were not evident in women (P > 0.501). **C**. Mean (SEM) for the postprandial insulin response in the *APOB* del/del (n = 10, open circles), del/ins (n = 15, open squares) and ins/ins (n = 10, open triangles) genotype groups after consumption of a test breakfast (49 g fat) at 0 min and a test lunch (29 g fat) at 330 min. There was a 69% higher AUC in the ins/ins than ins/del (P = 0.004) and del/del (P = 0.032) groups.

There was a significant effect of the *APOB* polymorphism on the postprandial TAG response (Figure 1A), with a 44% and 31% higher TAG AUC (del/del vs ins/del, P = 0.010; del/del vs ins/ins, P < 0.001; ins/del vs ins/ins, P < 0.001) and 47% and 36% higher IAUC (del/del vs ins/ins, P < 0.001; ins/del vs ins/ins, P = 0.001) in the ins/ins than del/del homozygotes and ins/del heterozygotes, respectively (Table 2). The polymorphism explained 35% and 20% of the variation in the TAG AUC and IAUC, respectively. The penetrance of genotype on TAG AUC and IAUC did not vary by age (P > 0.94) or BMI (P > 0.10); however, there was a significant interaction with gender (P < 0.04), with an impact of genotype observed only in men. Even after excluding the ins/ins individuals (n = 25), given the gender imbalance in this group (Table 2), a gender-specific effect of genotype on postprandial TAG AUC was still observed (men, P = 0.01; women, P = 0.24) (Figure 1B).

For the postprandial insulin response (Figure 1C), a significant effect of the polymorphism on AUC but not IAUC (P = 0.108) was observed, with a 69% higher AUC in the ins/ins than ins/del (P = 0.004) and del/del (P = 0.032) groups. The genotype explained 7% of the variation in AUC. Significant genotype effects were not observed for the summary measures of the postprandial glucose and NEFA responses.

### Discussion

Our data provides evidence of a significant role of the *APOB* ins/del polymorphism in determining postprandial TAG and insulin responses to sequential meal ingestion. Furthermore, we have shown an impact of this polymorphism on the fasting lipid profile, insulin and HOMA-IR, a surrogate measure of insulin resistance.

The ins/del polymorphism arises due to an insertion or a deletion of 9 base pairs that produces apoB signal peptides of 27 and 24 amino acids in length, respectively [21]. A less effective translocation of newly synthesised apoB across the endoplasmic reticulum membrane has been reported with the shorter signal peptide, suggesting this polymorphism may impact on TAG-rich lipoprotein (TRL, CM and VLDL) production. As with previous findings [8,9,19], insertion allele carriers showed higher fasting and postprandial TAG concentrations which was associated with decreased HDL-C levels. Intriguingly, the dose response for the impact of the insertion allele on TAG-AUC (ins/ins > ins/del > del/del) was only evident in men, a phenomenon we have previously observed in our cohort for the *LEPR* [20] and *APOA5* [22] genotypes. Expression of the biochemical phenotype of this *APOB* polymorphism has been proposed to be dependent on the population studied [19]. Irrespective of genotype, men had higher BMI, fasting TAG, insulin and HOMA-IR, and lower HDL-C than women (Table 1), which may have influenced the genotype-gender relationships with postprandial TAG handling. However, the small proportion of women in our dataset (n = 35/147) limits definite conclusions that the impact of genotype is only evident in men.

Dietary fat quantity has been reported to influence the effect of this genotype on TAG levels, with a greater association observed when participants consumed a high-fat than low-fat diet. Xu et al. [4] proposed the overall reduction in TRL synthesis during the low-fat diet to be responsible for the loss of the genotype effect on the intracellular handling of apoB. Furthermore, the TRL-TAG content, but not particle number (apoB concentration), was different between the ins/del groups during the high-fat diet indicating this polymorphism may also influence TRL composition. The reduced ability of the ins/ins homozygotes to handle dietary TAG after meal ingestion in the present study was associated with a higher fasting HOMA-IR and postprandial insulin AUC. In the absence of any known direct effect of apoB on insulin production or cellular action, it is likely that the higher insulin levels are in response to higher postprandial TAG. The associated loss of insulin sensitivity would likely impact on various stages of TRL metabolism. However, we did not determine apoB in the present study and hence it is difficult to discriminate between the potential contributions of increased TRL production versus impaired TAG clearance (both highly insulin dependent processes), to the higher TAG response in the insertion allele carriers.

In summary, our findings indicate the *APOB* ins/del polymorphism is likely to be an important determinant of the inter-individual variability in the postprandial TAG and insulin responses to dietary fat intake. However, we were unable to replicate these findings due to the lack of access to another postprandial cohort with data on this polymorphism. Hence, replication is highly warranted and, to determine in particular whether the penetrance of genotype on the TAG response is gender-specific. Further work is required to investigate the impact of the *APOB* ins/del polymorphism on TRL metabolism, and coronary heart disease risk.

## Abbreviations

ApoB: Apolipoprotein B
HDL-C: High density lipoprotein-cholesterol
HOMA-IR: Homeostasis assessment model of insulin resistance
LDL-C: Low density lipoprotein-cholesterol
NEFA: Non-esterified fatty acids
TAG: Triacylglycerol
TC: Total cholesterol
